# Rosiglitazone Protects Endothelial Cells From Irradiation-Induced Mitochondrial Dysfunction

**DOI:** 10.3389/fphar.2020.00268

**Published:** 2020-03-13

**Authors:** Bjorn Baselet, Ronald B. Driesen, Emma Coninx, Niels Belmans, Tom Sieprath, Ivo Lambrichts, Winnok H. De Vos, Sarah Baatout, Pierre Sonveaux, An Aerts

**Affiliations:** ^1^Institute for Environment, Health and Safety, Radiobiology Unit, Belgian Nuclear Research Centre (SCK CEN), Mol, Belgium; ^2^Institute of Experimental and Clinical Research (IREC), Pole of Pharmacology and Therapeutics, Université catholique de Louvain (UCLouvain), Brussels, Belgium; ^3^Laboratory of Morphology, Biomedical Research Institute (BIOMED), Hasselt University, Diepenbeek, Belgium; ^4^Neural Circuit Development and Regeneration Research Group, KU Leuven, Leuven, Belgium; ^5^Faculty of Medicine and Life Sciences, Biomedical Research Institute, Hasselt University, Hasselt, Belgium; ^6^Cell Systems and Imaging Research Group (CSI), Department of Molecular Biotechnology, Ghent University, Ghent, Belgium; ^7^Laboratory of Cell Biology and Histology, Department of Veterinary Sciences, University of Antwerp, Antwerp, Belgium; ^8^Department of Molecular Biotechnology, Ghent University, Ghent, Belgium

**Keywords:** ionizing radiation, endothelial cells, rosiglitazone, mitochondria, cardiovascular disease

## Abstract

**Background and Purpose:**

Up to 50–60% of all cancer patients receive radiotherapy as part of their treatment strategy. However, the mechanisms accounting for increased vascular risks after irradiation are not completely understood. Mitochondrial dysfunction has been identified as a potential cause of radiation-induced atherosclerosis.

**Materials and Methods:**

Assays for apoptosis, cellular metabolism, mitochondrial DNA content, functionality and morphology were used to compare the response of endothelial cells to a single 2 Gy dose of X-rays under basal conditions or after pharmacological treatments that either reduced (EtBr) or increased (rosiglitazone) mitochondrial content.

**Results:**

Exposure to ionizing radiation caused a persistent reduction in mitochondrial content of endothelial cells. Pharmacological reduction of mitochondrial DNA content rendered endothelial cells more vulnerable to radiation-induced apoptosis, whereas rosiglitazone treatment increased oxidative metabolism and redox state and decreased the levels of apoptosis after irradiation.

**Conclusion:**

Pre-existing mitochondrial damage sensitizes endothelial cells to ionizing radiation-induced mitochondrial dysfunction. Rosiglitazone protects endothelial cells from the detrimental effects of radiation exposure on mitochondrial metabolism and oxidative stress. Thus, our findings indicate that rosiglitazone may have potential value as prophylactic for radiation-induced atherosclerosis.

## Introduction

High radiation doses (≥2 Gy) are known to increase the risk of cardiovascular diseases (CVD) after radiotherapy (RT) (Hildebrandt, 2010; Shimizu et al., 2010; Darby et al., 2013). Given that 50 – 60% of all cancer patients receive RT in their treatment scheme (Gottfried et al., 1996), the excess risk of developing CVD is a societal concern. Yet, the vascular effects of ionizing radiation are not completely understood, as knowledge of the underlying biological and molecular mechanism of radiation-induced CVD is currently limited (Little et al., 2008; Hildebrandt, 2010), resulting in possibly inadequate radiation protection.

One of the targets of ionizing radiation that is linked to radiation-induced CVD is the endothelium (Little et al., 2008; Hildebrandt, 2010). Exposure of endothelial cells to radiation results in endothelial cell dysfunction (Bhattacharya and Asaithamby, 2016), promoting the development and progression of atherosclerosis (Vita and Keaney, 2002; Widmer and Lerman, 2014). Atherosclerosis is a complex process in which arteries narrow due to plaque formation, a process characterized by DNA damage, inflammation, senescence and apoptosis (Yu et al., 2012). Recent evidence indicates that mitochondria play a crucial role in the cellular and molecular mechanisms underlying atherosclerosis. In most cells mitochondria supply the bulk of cellular energy reserves through the process of oxidative phosphorylation (OXPHOS). Endothelial cells, however, are reported to be glycolytic and to minimally rely on mitochondria for ATP generation. Rather than providing energy, mitochondria in endothelial cells may act as signaling organelles that control cytosolic Ca^2+^ signaling or modify reactive oxygen species (ROS) (Wilson et al., 2019). While minute levels of ROS are crucial for cellular signaling pathways, an excess can lead to a state of oxidative stress, which may drive cells into senescence or trigger apoptosis (Yu et al., 2012; Sieprath et al., 2015). Likewise, dysregulation of cellular calcium homeostasis may lead to cell death.

In humans, mitochondrial dysfunction has been evidenced by the presence of mitochondrial DNA (mtDNA) damage in the vessel wall, in atherosclerotic plaques and in circulating leukocytes of patients affected by atherosclerosis (Corral-Debrinski et al., 1992; Bogliolo et al., 1999; Botto et al., 2005). Elevated human leukocytic mtDNA damage was also found to be indicative of a higher atherosclerosis risk (Yu et al., 2013). Experiments using ApoE^–/–^ mice which harbor a polymerase gamma mutation (that makes them more prone to accumulate mtDNA mutations) revealed that elevated aortic mtDNA damage was associated with an increased atherosclerotic plaque size, thus providing direct causal evidence (Yu et al., 2013). Moreover, a range of studies have demonstrated the presence of changes in mitochondrial number, structure and function after exposure of different types of cells and tissues to high (5–20 Gy) doses of ionizing radiation, indicating induction of mitochondrial dysfunction (Pandey et al., 2006; Nugent et al., 2007; Dayal et al., 2009; Hu et al., 2017; Lafargue et al., 2017). However, findings specifically focusing on the effect of ionizing radiation exposure on endothelial mitochondria are scarce. Therefore, we investigated (Darby et al., 2013) whether a single exposure to a therapeutically relevant single radiation dose induces mitochondrial dysfunction in endothelial cells, (Shimizu et al., 2010) how mitochondrial dysfunction (such as pre-existing in people with an increased atherosclerotic burden) can affect the endothelial cell response to ionizing radiation, and (Hildebrandt, 2010) whether treatment of endothelial cells with rosiglitazone, a peroxisome proliferator-activated receptor gamma (PPARγ) agonist, could help sustain mitochondrial function after exposure to ionizing radiation.

## Materials and Methods

### Cells and Irradiation

Human telomerase-immortalized coronary artery endothelial (TICAE) cells (from the European Collection of Authenticated Cell Cultures [ECACC]) were grown in Human MesoEndo Endothelial Cell Medium (Cell Applications catalog #212–500) and cultured at 37°C with 5% CO_2_ in a humidified incubator. Cells were irradiated at confluence with 2 Gy X-rays (dose rate of 0.50 Gy/min), using an Xstrahl RX generator (250 kV, 12 mA, 3.8 mm Al and 1.4 mm Cu). Sham-irradiated cells were used as control. Cells were not passaged after exposure. For lentiviral production, HEK-293T/17 cells (from American Type Culture Collection [ATCC]) were maintained in DMEM (Thermo Fisher Scientific) supplemented with 1% L-glutamine (Sigma-Aldrich), 10% fetal bovine serum, 1% penicillin/streptomycin, 1% non-essential amino acids, and 0.1% sodium pyruvate (Thermo Fisher Scientific). In order to induce mitochondrial dysfunction in endothelial cells, TICAE cells were grown in 50 ng/ml ethidium bromide (Sigma-Aldrich) for 7 weeks to yield TICAE-EtBr cells (King and Attardi, 1989). Other TICAE cells were treated with 50 μM PPARγ agonist rosiglitazone (Sigma-Aldrich) for their entire lifespan (TICAE-ROSI cells). Because mtDNA-damaged cells are auxotrophic for uridine and pyruvate (King and Attardi, 1989), TICAE-EtBr cells were kept in Human MesoEndo Endothelial Cell Medium (Cell Applications catalog #212–500) supplemented with 50 μg/ml uridine (Applichem), 2 mM L-glutamine, 100 μg/ml sodium pyruvate and 25 mM HEPES. TICAE-ROSI cells were cultured in non-supplemented Human MesoEndo Endothelial Cell Medium (Cell Applications catalog #212–500).

### Generation of Mito-GFP-Expressing Endothelial Cells

Lentiviral particles were produced in HEK-293T/17 cells with the LentiStarter 2.0 kit (System Biosciences) by transfecting 10 μg pCT-Mito-GFP-pCMV lentiviral construct (CYTO102-PA-1) along with 2 μg transfer plasmid, 20 μl pPACKH1 plasmid mix (Systems Biosciences) and 8 μg/ml polybrene (Sigma-Aldrich) per 10 cm-wide dish. At 18 h post transfection, fresh medium was added to cells. Virus-containing supernatants were collected 24 and 48 h later. Samples were combined, centrifuged, filtered through a 0.45-micron filter, aliquoted, and stored at −80°C. At 50% confluence, TICAE cells were transduced in Human MesoEndo Endothelial Cell Medium containing >5 × 10^5^ infectious units/ml. After 72 h, stable expressing cells were selected with 1 μg/ml puromycin.

### Mitochondrial DNA Copy Number Assay

Total DNA was extracted using the QIAamp DNA mini kit (Qiagen) with RNase treatment, according to the manufacturer’s instructions. To quantify mtDNA copy number, TaqMan RT-PCR was performed using a 7,500 Fast Real-Time PCR system (Applied Biosystems). TaqMan Universal Master Mix II (Thermo Fisher Scientific) was used according to the manufacturer’s instructions, with 1x TaqMan RNase P copy number reference (Thermo Fisher Scientific) and a mtDNA specific primer-probe combination that has been described elsewhere (forward primer: GTA-CCC-ACG-TAA-AGA-CGT-TAG-G; reverse primer: TAC-TGC-TAA-ATC-CAC-CTT-CG) (Pogozelski et al., 2003). The mtDNA content was defined as the ratio of mtDNA specific sequence to the single-copy human nuclear control gene RNase P (Thermo Fisher Scientific).

### Mitochondrial Content Measurement

Cells were plated at 10,000 cells/well in 96-well plates. After irradiation, cells were imaged at 10 × magnification in an IncuCyte Zoom Live-content imaging system (Essen Bioscience) at 37°C, 5% CO_2_. Images (2/well) were recorded every 2 h for 5 days. Data were analyzed using IncuCyte analysis software to detect and quantify mtGFP-expressing mitochondrial content/image. Results were normalized according to the degree of confluence as determined with phase contrast images.

### High Content Live Cell Imaging of Intracellular ROS and Mitochondrial Membrane Potential

Levels of intracellular ROS and mitochondrial membrane potential were measured as described elsewhere (Sieprath et al., 2016; Sieprath et al., 2017). Briefly, (sham-)irradiated endothelial cells were grown in 96-well plates and stained for 25 min at room temperature with 2 μM chloromethyl-2′,7′-dichlorodihydrofluorescein diacetate (CM-H_2_DCFDA) (Life Technologies) together with 100 nM tetramethyl rhodamine methyl ester (TMRM, Invitrogen). After washing in Hank’s balanced salt solution with 20 mM HEPES, all wells (4 fields per well) were imaged sequentially with a Nikon Ti Eclipse inverted wide field fluorescence microscope using a 20x air Plan Fluor objective (NA 0.50). After this first imaging cycle, 20 μM tert-butyl peroxide (tBHP, Sigma-Aldrich) was added and after 3 min the same acquisition cycle was repeated All images were processed with an automated image analysis script (RedoxMetrics.ijm) for FIJI (National Institutes of Health^1^, 1997–2014). This pipeline is based on a sequence of image processing steps including a flat field correction, noise reduction by Gaussian filtering, cell or mitochondrial segmentation and subsequent feature analysis of regions of interest. For CM-H_2_DCFDA, cell segmentation was performed by automatic thresholding according to Huang’s algorithm (Huang, 1989) and average intensities were measured within the segmented regions. For TMRM, mitochondrial signals were enhanced by local contrast enhancement and multi-scale Laplacian filtering, after which binarization was performed using Huang’s algorithm. Resulting masks were used to measure signal intensities of objects larger than three pixels on the original image.

### ATP Assay

Intracellular ATP levels were measured using the ATPlite 1step assay (Perkin Elmer) according to manufacturer’s instructions. Briefly, cells in a 96-well plate were lysed with ATPlite solution at different times after irradiation. After shaking (700 RPM, 2 min), luminescence signals were measured with a CLARIOstar microplate reader (BMG LABTECH).

### Oximetry

OCR levels were measured using a Cell Mito Stress Test Kit on a Seahorse XF96 analyzer (Agilent) according to manufacturer’s instructions. Briefly, cells were irradiated and plated at 20,000 cells/well in XF96-well plates. The next day, cells were washed and equilibrated with buffer-free medium (DMEM + Glutamax with 10 mM glucose and 2 mM L-glutamine) at 37°C in a CO_2_-free incubator for 1 h. Initial measurements of ECAR and OCR were collected. Cells were then consecutively treated with 1 μM oligomycin, 1 μM FCCP and 0.5 μM rotenone/antimycin A, during which OCR values were assessed. Data were normalized to cell numbers obtained by counting cell nuclei after paraformaldehyde fixation and DAPI staining.

### Apoptosis Assays

For caspase 3/7 activation and annexin V apoptosis assays, cells were plated at 6,000 cells/well in 96-well plates. Caspase 3/7 and annexin V green reagent (Essen Bioscience) was added at a 1:1,500 dilution. Cells were imaged at 10 × magnification on an IncuCyte Zoom Live-content imaging system (Essen Bioscience) at 37°C, 5% CO_2_. Two images/well were acquired every 2 h for 98 h. Data were analyzed using the IncuCyte analysis software. Results were normalized to total cell count determined with NucLight (Essen Bioscience), a nuclear label for living cells. The apoptotic index was calculated by dividing the final apoptotic cell count by the total cell count per irradiation group (either 0 or 2 Gy) and then dividing the respective values of 2 Gy by those of the 0 Gy group.

### Transmission Electron Microscope (TEM) Imaging

Cells were seeded on Thermanox slides (Nunc). After irradiation, cells were fixed with 2% glutaraldehyde in 0.05 M cacodylate buffer (pH 7.3) at 4°C. The fixative was removed, and samples were post-fixed in 2% osmium tetroxide for 1 h at room temperature, followed by staining with 2% uranyl-acetate in 10% acetone for 20 min at room temperature. Samples were gradually dehydrated by a series of graded acetone concentrations and embedded in epoxy resin (Araldite). Ultra-thin sections (0.06 μm thick) were mounted on 0.7% formvar coated grids (Agar scientific), contrasted with uranyl-acetate followed by lead citrate, and examined on a Philips EM 208 transmission electron microscope operating at 80 kV.

### Statistics

Data show means ± SEM. Data were analyzed using GraphPad v7.0.2. Data from mtGFP, mitochondrial OXPHOS, ATP, oxidative stress and TMRM measurements were analyzed by two-way ANOVA with Bonferroni *post hoc* test. Data from cellular mtDNA content and cell death measurements were analyzed by one-way ANOVA with Tukey *post hoc* test. *P* < 0.05 was considered to be statistically significant.

## Results

### Irradiated Endothelial Cells Have a Persistently Low Mitochondrial Number

**FIGURE 1:**
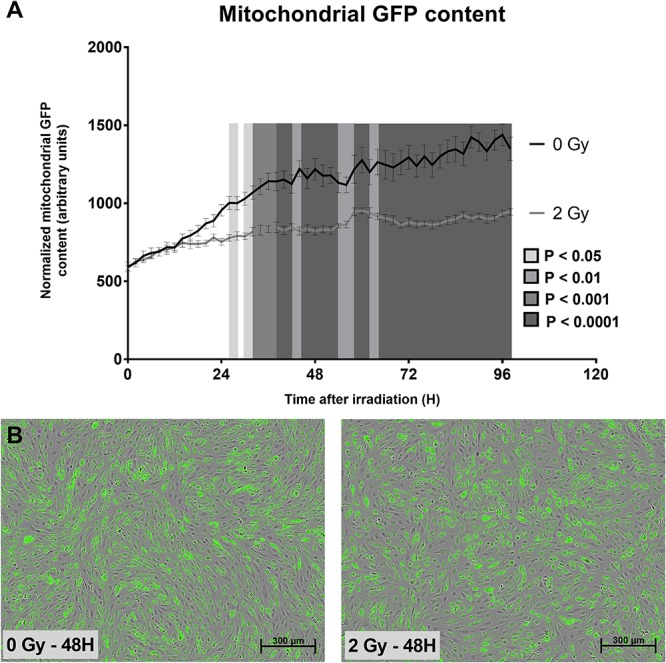
Irradiation decreases TICAE cell mitochondrial abundance. **(A)** The graph shows mitochondrial GFP content per cell over time in sham-irradiated and 2 Gy-irradiated TICAE cells, determined using live cell imaging and normalized according to masked cell count based on bright field images. *P*-values are indicated as colored blocks (*N* = 3, *n* = 12). **(B)** Representative images of 0 Gy 48 h after irradiation (left) and a 2 Gy 48 h after irradiation (right). Bar = 300 μm. Statistics were performed by a multiple comparison two-way ANOVA with Bonferroni *post hoc* test.

To gain more knowledge about the mitochondrial effects of X-ray irradiation on endothelial cells, we first tested whether irradiation of TICAE cells (Figure 1A) constitutively expressing mtGFP (a marker of mitochondrial content) with a single 2 Gy X-ray dose induced changes in mitochondrial number during a time span of 98 h after ionizing radiation exposure. We found that the mGFP signal, normalized according to the degree of confluence as determined with phase contrast images, increased at a slower rate in 2 Gy-irradiated compared to sham-irradiated TICAE cells over time, with “time after irradiation,” “radiation dose” and their interaction being significantly different (*P* < 0.0001; Figures 1A,B).

### More Abundant Mitochondria Decrease Apoptosis in Irradiated Endothelial Cells

**FIGURE 2:**
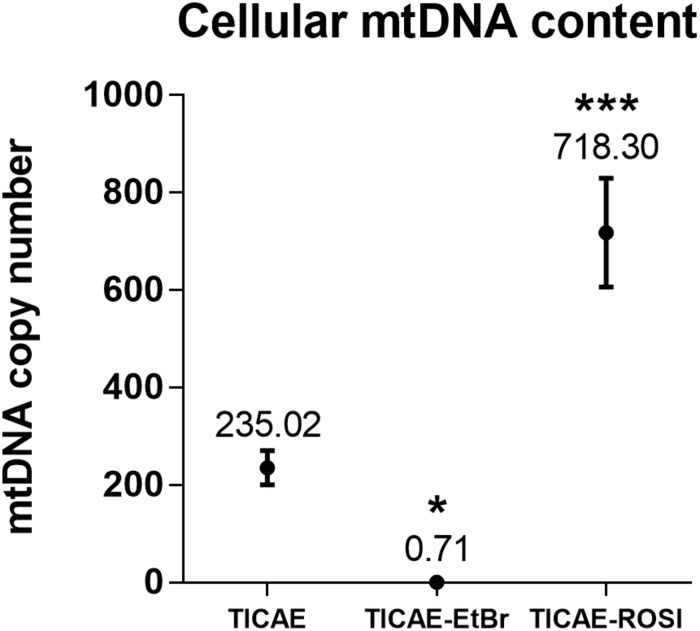
Validation of TICAE-EtBr and TICAE-ROSI models. mtDNA copy number in untreated TICAE cells (control) and in TICAE-EtBr and TICAE-ROSI cells (*N* = 3, *n* = 12). *P < 0.05 and ***P < 0.001 by one-way ANOVA with Tukey *post hoc* test.

To study how preexisting mitochondrial dysfunction can affect TICAE cell response to ionizing radiation exposure, cells were treated either chronically with ethidium bromide to generate TICAE-EtBr cells with mitochondrial dysfunction (King and Attardi, 1989) or with rosiglitazone to increase mitochondrial function in TICAE-ROSI cells (Wilson-Fritch et al., 2003). mtDNA copy number was assessed relative to nuclear DNA. Untreated TICAE cells had an average of 235.02 ± 35.00 mtDNA copies per cell (Figure 2). In comparison, TICAE-EtBr cells had a significantly diminished amount of mtDNA copies per cell (0.71 ± 0.17) and TICAE-ROSI cells a significantly elevated amount of mtDNA copies per cell (718.30 ± 111.68).

**FIGURE 3:**
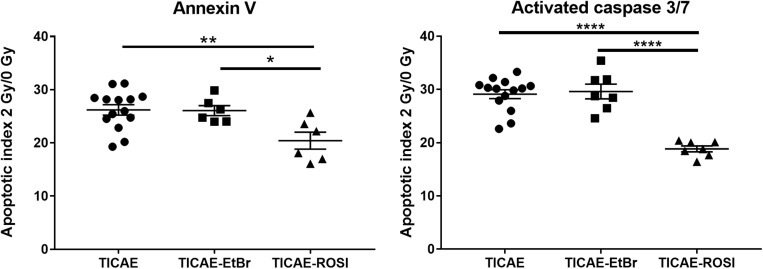
Rosiglitazone treatment protects TICAE cells against irradiation. Graphs show the apoptotic index of TICAE, TICAE-EtBr and TICAE-ROSI cells 98 h after a single 2 Gy irradiation, using annexin V (*left*) and activated caspase 3/7 staining (*right*) (*N* = 2, *n* = 7–14). *P < 0.05, **P < 0.01, ****P < 0.0001 by one-way ANOVA with Tukey *post hoc* test.

We then compared the response of the cells to irradiation. Following a 2 Gy X-ray dose, a high amount of apoptotic TICAE cells was observed (Figure 3). TICAE-EtBr responded with the same amplitude. Comparatively, TICAE-ROSI cells had significantly lower apoptotic indexes based on either annexin V content (20.42 ± 1.59) or caspase 3/7 activity (18,83 ± 0.55) than TICAE cells (26.20 ± 0.97 for annexin V and 29.11 ± 0.83 for caspase 3/7 activity) and TICAE-EtBr cells, (respectively, 26.07 ± 0.94 and 29.61 ± 1.38).

### Irradiation Decreases Endothelial Cell Respiration but Increases Their Oxidative Capacity

**FIGURE 4:**
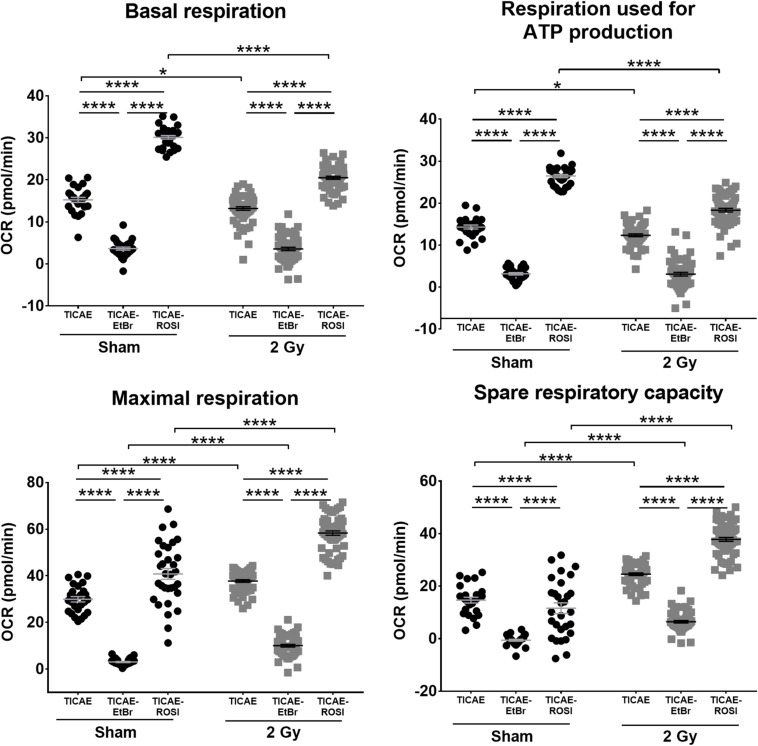
Irradiation decreases TICAE cell respiration but increases their respiration capacity. The rates of basal respiration, respiration linked to ATP production, maximal respiration and spare respiratory capacity were measured using Seahorse oximetry in TICAE, TICAE-EtBr and TICAE-ROSI cells 24 h after sham (●) or 2 Gy X-ray (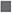) exposure (*N* = 3, *n* = 32–64). *P < 0.05, ****P < 0.0001 by two-way ANOVA with Bonferroni *post hoc* test.

mtDNA is not protected by histones and could be a primary target of ionizing radiation in endothelial cells. Using oximetry, we therefore tested whether irradiation could impact mitochondrial respiration in our cell models. As expected, basal respiration, respiration used for ATP production, maximal respiration and spare respiratory capacity were all lower in sham-irradiated TICAE-EtBr and all higher in TICAE-ROSI compared to TICAE cells (Figure 4). In TICAE cells, a single 2 Gy X-ray exposure significantly decreased basal respiration and respiration used for ATP production, whereas it increased maximal respiration and the spare capacity measured 24 h later. Compared to sham, irradiated TICAE-EtBr had unchanged basal respiration and respiration used for ATP production, but maximal respiration and spare capacity slightly increased, although with a much lower amplitude compared to TICAE cells, indicating that TICAE-EtBr were not totally devoid of mitochondria. Irradiated TICAE-ROSI cells responded to irradiation in the same way as TICAE cells, but changes were of a larger amplitude. Thus, irradiation decreased actual respiration rates of the cells but increased their potential to respire.

### Irradiation Minimally Affects the Intracellular ATP Content of Endothelial Cells

**FIGURE 5:**
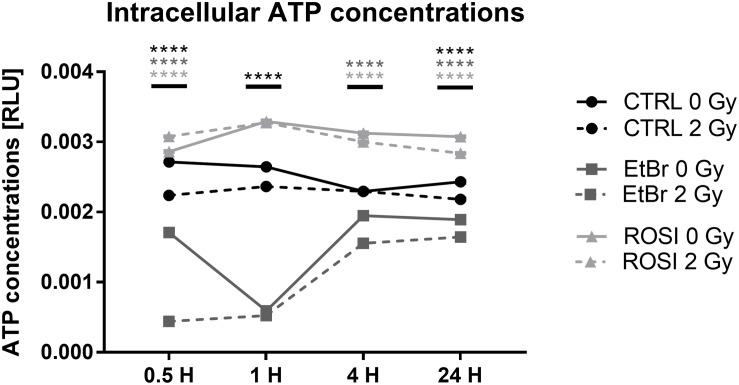
Irradiation minimally affects ATP levels in endothelial cells. Measurements of ATP concentrations in TICAE, TICAE-EtBr and TICAE-ROSI cells at increasing times after a sham or a 2 Gy X-ray irradiation (*N* = 2, *n* = 24). ****P < 0.0001 using two-way ANOVA with Bonferroni *post hoc* test.

Endothelial cells produce most of their ATP through glycolysis (De Bock et al., 2013). Still, the decreased oxygen consumption rates that we observed after irradiation could potentially impact intracellular ATP levels. We therefore analyzed ATP content over time. In sham-irradiated conditions, TICAE and TICAE-ROSI cells had stable ATP levels, which were significantly higher in TICAE-ROSI (Figure 5). TICAE-EtBr had lower ATP levels, which nevertheless progressively increased over time, probably reflecting a higher glycolytic activity, as previously shown in cancer cells (Sonveaux et al., 2008). Irradiation minimally but statistically significantly impacted ATP levels in all 3 cell lines with “time after irradiation,” “radiation dose” and their interaction being significantly different (all *P* < 0.0001; except “radiation dose” TICAE-ROSI: *P* < 0.05).

### Mitochondria Control Redox Stress in Endothelial Cells After Irradiation

**FIGURE 6:**
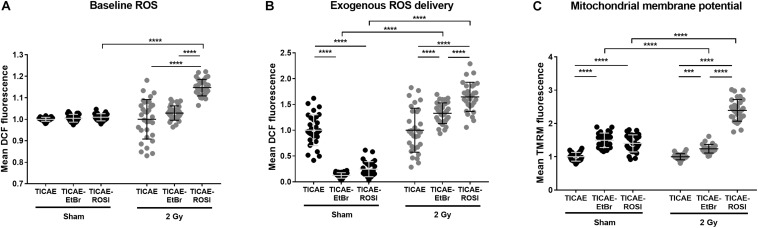
Endothelial cells with dysfunctional mitochondria have difficulties to cope with redox stress upon irradiation. **(A)** Intracellular ROS measurements in TICAE, TICAE-EtBr and TICAE-ROSI cells 24 h after a sham or a 2 Gy X-ray irradiation (*N* = 3, *n* = 32). **(B)** Same as A but basal ROS were measured after exposing the cells to 20 μM tert-butyl peroxide (tBHP) (*N* = 3, *n* = 32). **(C)** Mitochondrial membrane potential of TICAE, TICAE-EtBr and TICAE-ROSI cells 24 h after a sham or a 2 Gy X-ray irradiation (*N* = 3, *n* = 32). ***P < 0.005, ****P < 0.0001 by two-way ANOVA with Bonferroni *post hoc* test.

Dysfunctional mitochondria are well known to have altered mtROS production (Gutterman, 2005; Kumari et al., 2014). In endothelial cells, intracellular ROS levels were similar in sham-irradiated TICAE, TICAE-EtBr, and TICAE-ROSI cells (Figure 6A). A single 2 Gy X-ray irradiation increased intercellular ROS content 24 h after irradiation only in TICAE-ROSI cells, which also displayed the highest oxidative capacity (see Figure 4).

We also tested resistance to redox stress by measuring intracellular ROS levels after challenging the cells with tBHP. TICAE-EtBr and TICAE-ROSI cells were more efficient to inactivate exogenously delivered ROS compared to TICAE (Figure 6B). However, the opposite was observed when performing tBHP treatment 24 h after irradiation, indicating that TICAE-EtBr and TICAE-ROSI exhaust their antioxidant defense systems faster than TICAE cells. Accordingly, TICAE-EtBr and TICAE-ROSI had a higher mitochondrial potential compared to TICAE cells, both in sham and irradiated conditions, (Figure 6C), which is generally associated with a high mitochondria-generated redox stress (Korshunov et al., 1997; Turrens, 2003).

### Irradiation Triggers Mitochondrial Remodeling in Endothelial Cells

**FIGURE 7:**
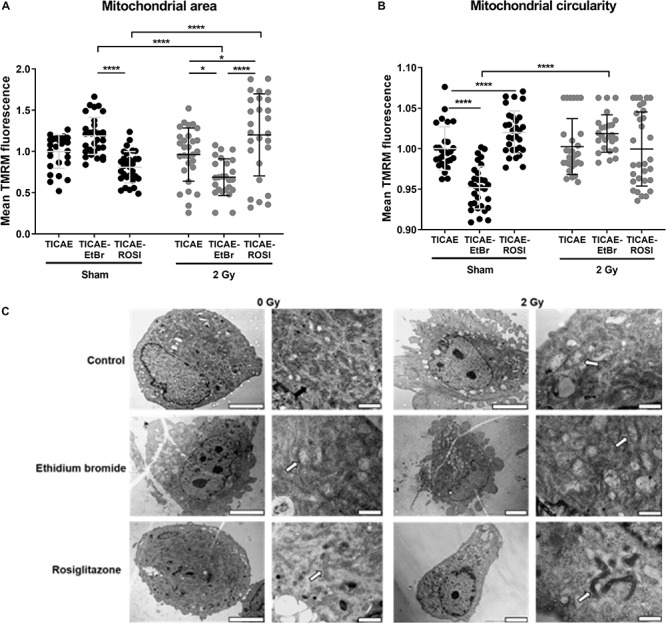
Irradiation alters mitochondrial morphology in endothelial cells. **(A)** Mitochondrial surface relative to cell surface was measured in TMRM-stained TICAE, TICAE-EtBr and TICAE-ROSI cells 24 h after a sham or a 2 Gy X-ray irradiation (*N* = 3, *n* = 32). **(B)** As in A, but measuring mitochondrial circularity (*N* = 3, *n* = 32). **(C)** Representative TEM pictures of TICAE, TICAE-EtBr and TICAE-ROSI cells 24 h after a sham or a 2 Gy X-ray irradiation. Black arrows point at normal mitochondria and white arrows at abnormal mitochondria. Bars = 5 μm. *P < 0.05, ****P < 0.0001 by two-way ANOVA with Bonferroni *post hoc* test.

To associate mitochondrial dysfunction to potential alteration of mitochondrial morphology, we first examined mitochondrial area and circularity after TMRM staining. Compared to TICAE, TICAE-EtBr cells had an increased mitochondrial area per cell with qualitatively less circular mitochondria in the sham condition, but an increased mitochondrial area with normal circularity 24 h after 2 Gy X-ray-irradiation (Figures 7A,B). TICAE and TICAE-ROSI cells had similar mitochondrial areas per cell in the sham condition, but it increased in irradiated TICAE-ROSI cells. Mitochondrial circularity was statistically different between the cell types and treatment conditions, of which high mitochondrial circularity is associated with a high redox stress state (Ahmad et al., 2013; Hung et al., 2018).

Ultrastructural characterization was performed on TEM images (Figure 7C). Sham-irradiated TICAE cells showed a regular aligned plasma membrane and a nucleus with normally distributed heterochromatin. Mitochondria were distributed throughout the cytoplasm with well-organized cristae. Comparatively, sham-irradiated TICAE-EtBr cells showed spreading and blebbing of the plasma membrane. Their mitochondria were swollen and showed disruption of the cristae. TICAE-ROSI cells were represented by cells with either a regular aligned plasma membrane and or with shedding of small cytoplasmic vesicles. The majority of the mitochondria had a small size, elongated phenotype, and a minority showed disrupted cristae. Twenty-four hours after irradiation, TICAE and TICAE-EtBr cells were characterized by plasma membrane spreading and blebbing and swollen mitochondria with disrupted cristae. TICAE-ROSI cells responded differently, displaying a normal, aligned plasma membrane with small elongated mitochondria and a dense matrix.

## Discussion

Our study aimed at investigating (Darby et al., 2013) whether irradiation with a therapeutically relevant dose of X-rays induces mitochondrial dysfunction in endothelial cells, (Shimizu et al., 2010) how endothelial cells with preexisting mitochondrial dysfunctions respond to ionizing radiation exposure, and (Hildebrandt, 2010) whether rosiglitazone can prevent irradiation-induced mitochondrial dysfunctions in endothelial cells.

In respect to the first aim, we report that TICAE cell irradiation promotes apoptosis and induces mitochondrial dysfunction as shown by reduction in basal respiration and lowered ATP synthesis. However, OXPHOS capacity was intact, as substantiated by increased maximal respiration and spare respiratory capacity. In the same line, a recent study in fibroblasts showed a correlation between decreased basal respiration and apoptosis with normal maximal respiration (Kandel et al., 2016; Jang et al., 2017). The authors coupled the reduction in basal respiration to a disarrangement of the cytoskeleton that inhibited mitochondrial attachment. Similarly, ultrastructural analysis of TICAE cells after irradiation showed a disorganization of the cellular architecture with the presence of mitochondrial swelling and cristae disruption. Hu et al. (2017) previously demonstrated that a 5–20 Gy γ-ray-irradiation of human umbilical vein endothelial cells (HUVEC) induced apoptosis together with increased ROS production and decreased mitochondrial membrane potential 24 h after exposure. In contrast, our study reveals no changes in mitochondrial membrane potential, ROS levels and redox defenses. This could be due to the lower ionizing radiation dose of 2 Gy that we used, as ROS production in the work of Hu et al. (2017) was found to be dose-dependent. From a pathophysiological standpoint, mitochondrial dysfunction can induce atherosclerosis, as mentioned in the introduction. Thus, the mitochondrial dysfunction that we observed in irradiated TICAE cells could potentially activate molecular pathways underlying radiation-induced atherosclerosis observed in exposed individuals (Shimizu et al., 2010; Darby et al., 2013), which warrants further investigation.

Our second aim was to determine whether a preexisting mitochondrial dysfunction could alter the response of endothelial cells to ionizing radiation. To address this question, we generated TICAE-EtBr and TICAE-ROSI cells. As expected, compared to TICAE, non-irradiated TICAE-EtBr cells had decreased basal and maximal respiratory rates, diminished ATP production and an abnormal mitochondrial morphology. They had improved redox stress resistance in basal conditions. Upon irradiation, TICAE-EtBr cells showed a further deterioration characterized by the inability to detoxify intracellular ROS in the presence of an exogenous peroxide and affected (albeit minimally) ATP production kinetics. Reduced redox resistance could be explained by the advanced disintegration of the mitochondrial network after irradiation, of which important characteristics are mitochondrial swelling (that we evidenced using TEM and fluorescent microscopy), a reduction of the number of mitochondria, a decrease in mitochondrial area and diminished mitochondrial membrane potential. From these data, we conclude that irradiation worsens mitochondrial function in our TICAE-EtBr endothelial cells with a preexisting mitochondrial dysfunction. Based on our results, we propose that the mitochondrial dysfunction that is already present in established atherosclerotic plaques (Bogliolo et al., 1999) could be aggravated by ionizing radiation exposure in clinical settings. If this hypothesis was true, then irradiation could accelerate and worsen atherosclerosis outcome, which deserves experimental evaluation.

Our third aim was to determine whether endothelial cells could be protected against irradiation-induced mitochondrial dysfunction. To address this point, we chronically treated TICAE cells with rosiglitazone. This compound is a member of the thiazolidinedione family that activates peroxisome proliferator-activated receptor γ coactivator- α1 (PGC-1α), promoting mitochondrial biogenesis in adipose tissue (Wilson-Fritch et al., 2003) and HUVECs (Fujisawa et al., 2009). Other studies confirmed that activation of PGC-1α by rosiglitazone stimulates mitochondrial biogenesis and OXPHOS (Pardo et al., 2011; Zhang et al., 2018). In addition, rosiglitazone has radioprotective properties, as it is able to protect 12 Gy-irradiated mice from intestinal toxicity by reversing the inflammatory and apoptotic changes induced by radiation (Mangoni et al., 2017). Compared to TICAE, non-irradiated TICAE-ROSI cells had improved mitochondrial function, as evidenced by an elevated basal and maximal respiratory capacity, increased spare respiratory capacity and a marked increase in ATP production. The higher respiratory capacity of TICAE-ROSI cells was associated with the presence of elongated mitochondria, suggesting a process of mitochondrial adaptive remodeling. When delivering exogenous ROS, intracellular ROS levels were even decreased compared to TICAE cells, suggesting an intact ROS scavenging system. These data are in line with a previous study (Karnewar et al., 2016) in which treatment of human aortic endothelial cells with mitochondria-targeted esculetin, a member of the coumarin family with anti-inflammatory and anti-oxidant properties, resulted in a similar increase in respiratory capacity and resistance to oxidative stress. Other studies confirmed the upregulation of PGC-1α expression by ROSI, as it has been shown to promote mitochondrial biogenesis and to maintain OXPHOS (Pardo et al., 2011; Zhang et al., 2018). Upon irradiation, compared to TICAE, TICAE-ROSI cells showed an increased respiration capacity, enhanced respiration used for ATP production and decreased apoptosis. However, TICAE-ROSI behaved as TICAE-EtBr cells when exposed to oxidative stress, thus undergoing faster exhaustion of redox defenses than TICAE cells. Despite limited redox defenses, mitochondrial dysfunction and apoptosis were limited after irradiation. This might be due to an adaptive response protecting TICAE-ROSI cells against an overflow of ROS, as was observed in other cells (Belikova et al., 2009) and animals (Bakkal et al., 2013). Therefore, we conclude that rosiglitazone improved mitochondrial function in our model and protects against irradiation-induced apoptosis.

## Conclusion

We report that a single 2 Gy X-ray dose induces mitochondrial dysfunction in endothelial cells. Furthermore, TICAE-EtBr cells demonstrated a diminished mitochondrial function and a higher oxidative stress state after radiation exposure. TICAE-ROSI cells revealed enhanced mitochondrial function, resulting in a higher oxidative stress state, but an overall decrease in apoptosis after irradiation potentially due to preconditioning. Future research should look into therapeutically relevant fractionated radiation schemes to fully grasp the scope of the role of endothelial mitochondria in radiation-induced cardiovascular diseases. Other open questions that need to be resolved are the effect of ionizing radiation on mtDNA, mitochondrial fission, fusion, redox balance, mitophagy and the interplay with rosiglitazone treatment and PGC-1α in endothelial cells. As a result, improved understanding of radiation-induced cardiovascular diseases would prove valuable for the protection of patients exposed to ionizing radiation.

## Data Availability Statement

The raw data supporting the conclusions of this article will be made available by the authors, without undue reservation, to any qualified researcher.

## Author Contributions

BB took part during all experiments performed within the scope of this manuscript. Generation of endothelial cell lines, mitochondrial DNA copy number assay, ATP assays and apoptosis assays were supported by EC and NB. High content live cell imaging was supported by TS and WD. Transmission electron microscope imaging was supported by RD and IL. All authors helped with data interpretation, scientific guidance and preparation of the manuscript.

## Conflict of Interest

The authors declare that the research was conducted in the absence of any commercial or financial relationships that could be construed as a potential conflict of interest.

## Footnotes

1 http://rsb.info.nih.gov/ij/
